# Will ChatGPT-4 improve the quality of medical abstracts?

**DOI:** 10.1093/pch/pxae062

**Published:** 2024-09-12

**Authors:** Jocelyn Gravel, Chloé Dion, Mandana Fadaei Kermani, Sarah Mousseau, Esli Osmanlliu

**Affiliations:** Department of Pediatric Emergency Medicine, CHU Sainte-Justine, Université de Montréal, Montréal, Québec; Faculté de médecine, Université de Montréal, Montréal, Québec; Faculté de médecine, Université de Montréal, Montréal, Québec; Department of Pediatric Emergency Medicine, CHU Sainte-Justine, Université de Montréal, Montréal, Québec; Division of Pediatric Emergency Medicine, Montreal Children’s Hospital, McGill University, Montréal, Québec

**Keywords:** ChatGPT, Medical informatics, Editing

## Abstract

**Background:**

ChatGPT received attention for medical writing. Our objective was to evaluate whether ChatGPT 4.0 could improve the quality of abstracts submitted to a medical conference by clinical researchers.

**Methods:**

This was an experimental study involving 24 international researchers (the participants) who provided one original abstract intended for submission at the 2024 Pediatric Academic Society (PAS) conference. We asked ChatGPT-4 to improve the quality of the abstract while adhering to PAS submission guidelines. Participants received the revised version and were tasked with creating a final abstract. The quality of each version (original, ChatGPT and final) was evaluated by the participants themselves using a numeric scale (0–100). Additionally, three co-investigators assessed abstracts blinded to the version. The primary analysis focused on the mean difference in scores between the final and original abstracts.

**Results:**

Abstract quality varied between the three versions with mean scores of 82, 65 and 90 for the original, ChatGPT and final versions, respectively. Overall, the final version displayed significantly improved quality compared to the original (mean difference 8.0 points; 95% CI: 5.6–10.3). Independent ratings by the co-investigators confirmed statistically significant improvements (mean difference 1.10 points; 95% CI: 0.54–1.66). Participants identified minor (n = 10) and major (n = 3) factual errors in ChatGPT’s abstracts.

**Conclusion:**

ChatGPT 4.0 does not produce abstracts of better quality than the one crafted by researchers but it offers suggestions to help them improve their abstracts. It may be more useful for researchers encountering challenges in abstract generation due to limited experience or language barriers.

## INTRODUCTION

Natural language processing (NLP) is a field of artificial intelligence that focuses on the interaction between computers and humans using natural language (1). Large language models (LLM) refer to a breakthrough in the field of NLP, distinguished by their large size and pre-training on extensive datasets to acquire a broad understanding of language patterns (2). ChatGPT is an LLM-enabled chatbot able to produce text in response to multiple types of inputs (3). Since its launch in November 2022, scientific articles partially written by ChatGPT have been published (4–6). While many authors have acknowledged that ChatGPT can write scientific articles of adequate quality (7–13) many journals questioned the ethical aspects of using chatbots for scientific writing (14–18). Researchers identified important limitations of using ChatGPT for writing, including many demonstrations that commonly provide inaccurate references (19–21).

Many articles reported that ChatGPT can write credible scientific abstracts (22,23), but few studies rigorously assessed the quality of the abstracts written by ChatGPT. Gao et al. compared 50 abstracts from real publications to abstracts generated by ChatGPT and concluded that “ChatGPT writes believable scientific abstracts” (24). On the other hand, Ali and Singh advised that abstracts written by ChatGPT must be verified to detect self-additions (25).

To our knowledge, no study has quantitatively evaluated the quality of abstracts provided by ChatGPT and its potential for helping researchers improve their work. Our main objective was to evaluate whether ChatGPT could improve the quality of abstracts submitted to conferences by clinical researchers.

## METHODS

This was an experimental study conducted in October 2023 evaluating the benefit of asking ChatGPT (Version 4.0 OpenAI Inc, San Francisco, CA, USA) to revise abstracts submitted to a medical scientific conference.

A convenience sample of researchers (the participants) was invited to provide one abstract that they expected to submit to the 2024 Pediatric Academic Societies (PAS) conference. The participants were identified based on their experience of publication and previous participation in the PAS conference. They were acquaintances of one of the co-investigators and were personally invited by email. The deadline for submission to the PAS conference was October 31, 2023. Participants were first contacted in September to be informed of the study coming and in October for recruitment. We requested that they provide what they would consider their final version of the abstract. The deadline for participating was October 29.

The intervention of interest was the use of ChatGPT-4 to improve the abstract. To do this, we created the following prompt asking ChatGPT-4 to improve the quality of the abstract while adhering PAS submission guidelines: “Using the following guidelines: 1. Have a title; 2. Have an abstract containing the following five sections: introduction, objective, method, results and conclusion; have a maximum of 2600 characters (with space excluded title), improve the following scientific abstract for clarity: **insert abstract**.” Each abstract constructed by ChatGPT was returned to its corresponding participants within 24 hours of the reception of the original abstract. Participants were then invited to provide a final abstract using either nothing, parts of or the entire ChatGPT version to improve their abstract. It was expected that the participants would complete their final version following the reception of the ChatGPT version. In the end, there were three versions of each abstract:

1. **Original version**: The first version of the abstract initially submitted by the researcher.
2. **ChatGPT version**: The abstract constructed by ChatGPT using the prompt asking to improve the original version.
3. **Final version:** The version that the participant used at the end of the process.

The primary outcome was the quality of the abstract measured on a verbal numeric scale from 0 to 100, using the following instruction: “On a scale of 0 to 100% on the quality of the abstract, where 0% means poor quality and 100% the best possible abstract, how would you rate the abstract?” This evaluation was initially conducted by the participants considering they are the experts in the field of their abstract and because our primary outcome was to evaluate if ChatGPT could help researchers. In that way, their opinion is the most important. Multiple secondary outcomes were measured. Among them, the quality of the original and final versions of the abstracts was evaluated by three members of the research team (the co-investigators) using the same verbal numeric scale. This evaluation was blinded to the version (original vs. final). Moreover, participants identified whether the abstract fulfilled all requirements of the PAS conference, and the presence of any factual error. These errors were classified as minor (no impact on the conclusion of the abstract or minimal impact on the probability of acceptance of the abstract by the conference reviewers) or major. Participants also reported whether they considered that the use of ChatGPT improved the quality of their abstract (yes/no), and if it improved their chance of being accepted (yes/no). Participants were asked if the ChatGPT version could have been submitted as is. Reasons justifying the last answer were collected.

The independent variables collected as potential factors associated to the outcomes were related to the writer of the abstract (number of years as researcher, number of abstracts previously written, previous use of ChatGPT, fluidity in English) and the abstract (qualitative vs. quantitative study, presence of a table or a figure).

### Data analysis

The primary analysis was the mean difference in scores between the final and the original version of the abstract according to the participants. To do this, we calculated the difference (final abstract score minus original abstract score) for each abstract and reported the mean and 95% confidence interval (95% CI) for these differences. Other analyses included the mean score for each version (original, ChatGPT and final) and differences according to the score assigned by the three co-investigators.

The proportion of participants responding that the use of ChatGPT improved their abstract, and the number of abstracts in which a factual error was found were calculated. Finally, participants reported their final evaluation of the usefulness of the revision provided by ChatGPT.

An exploratory analysis was carried out to evaluate factors associated with the positive impact of the use of ChatGPT. To do this, we evaluated the association between independent variables related to the researchers (age group, gender, experience with abstract submission and ChatGPT) and to the abstracts (type, presence of figure), and improvement in score using ANOVA.

We had no prespecified idea of the mean scores for the evaluation of the abstracts. However, it was expected that the final abstract should not have a lower score than the original one as they are both written and evaluated by the same researcher who should improve or keep the same abstract. Based on our previous personal experience and after informal discussion with about ten researchers who have used ChatGPT, we anticipated small but constant improvement in the quality of abstracts after using ChatGPT. Expecting a constant improvement, we anticipated a large effect size. A common value reported to represent large standardized effect size is 0.8 (26). Being conservative and using a standardized effect size of 0.7, it was estimated that we needed to evaluate at least 20 abstracts.

Our local Institutional Review Board concluded that no ethical review was required for the project as researchers are viewed as raters and not participants.

## RESULTS

A total of 33 researchers were directly invited to collaborate in the study. Among them, six did not plan to submit an abstract, one did not respond and one was not at ease to use ChatGPT for abstract generation. In the end, 25 participants provided an abstract and 24 (96%) completed the evaluation of their three abstracts.

Table 1 provides a comprehensive overview of the participants and abstracts engaged in the study. In brief, 15 studies (63%) employed quantitative methodologies and 7 studies (30%) incorporated tables or figures to enhance data representation. The participants group exhibited notable diversity, with 50% of participants being under the age of 34, 58% identifying as female and 50% boasting a track record of more than 15 published abstracts. Regarding ChatGPT usage, 42% of participants had never utilized the tool, 50% had used it sporadically and 8% employed it regularly. Proficiency in English was evenly distributed across various comfort levels, with eight (33%) participants reporting their proficiency as moderately, quite or very comfortable, respectively.

**Table 1.** Characteristics of the abstracts and participants (n = 24)

	N (%)
Type of study	
Quantitative	15 (63)
Qualitative	5 (21)
Other	4 (17)
Presence of table/figure	7 (30)
Participants’ information	
Age group	
24 years	3 (13)
34 years	9 (38)
44 years	7 (30)
54 years	4 (17)
64 years	1 (4)
Gender	
Female	14 (58)
Male	10 (42)
Number of previous abstracts	
This is the first	1 (4)
1–5	7 (30)
6–15	4 (17)
15	12 (50)
ChatGPT previous experience	
Never used it	10 (42)
Used it a few times	12 (50)
Used it many times	2(8)
English literacy	
Moderately comfortable	8 (33)
Quite comfortable	8 (33)
Very comfortable	8 (33)

**Figure 1.**
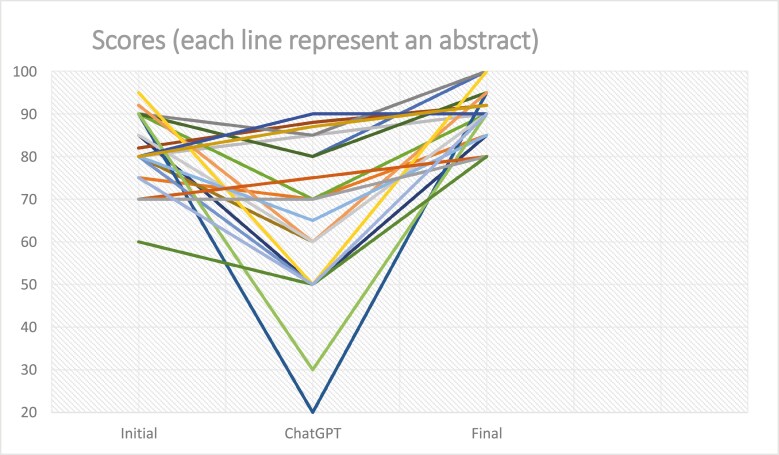
Scores of the initial, ChatGPT and final versions of the abstracts

Abstract quality varied between the three versions with mean scores of 82 (95% CI: 78–86), 65 (95% CI: 58–73) and 90 (95% CI: 88–93) for the original, ChatGPT and final versions, respectively. Overall, the final abstracts displayed significantly improved scores compared to the original ones according to the participants with improvement varying between 1 and 20 points (mean difference 8.0 points; 95% CI: 5.6–10.3). Of note, only three (14%) abstract scores remained unchanged in the final version. Figure 1 shows that for most series of abstract, the ChatGPT version had the lowest score, but the final version had a score higher than the original.

Participants identified minor (n = 10) and major (n = 3) factual errors in the abstracts generated by ChatGPT. Examples of major errors included omission of providing the primary outcome in the method section or confusion/omission in the results provided. Among the minor errors, ChatGPT omitted to provide confidence intervals in many abstracts. Eighteen (75%) participants reported that ChatGPT contributed to the enhancement of their final abstract and 10 (42%) participants believed it improved their abstract’s probability of acceptance. Finally, 18 (75%) participants reported being uncomfortable using the ChatGPT version for conference submission; mostly because of the omission of important information.

**Figure 2.**
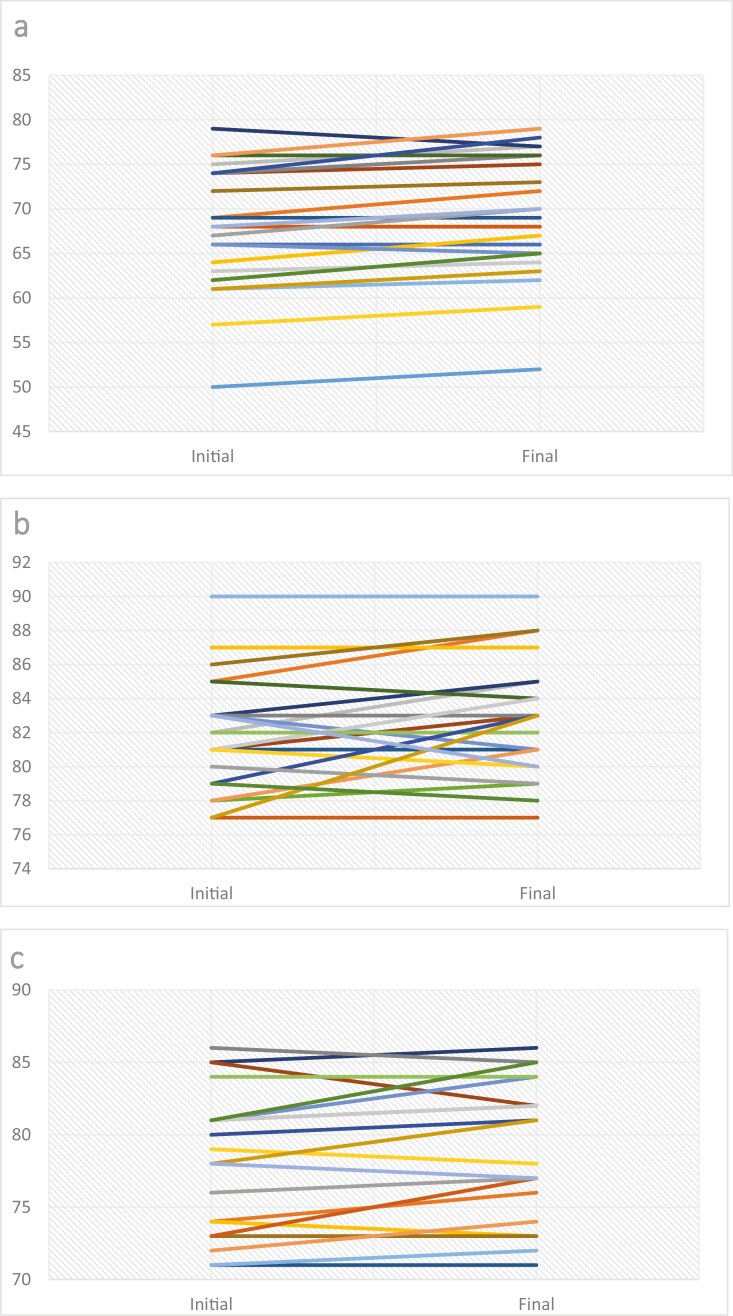
Individual scores for the initial and final abstracts provided by each rater (a, b and c)

All 24 pairs of abstracts (original and final) were evaluated in triplicate by co-investigators. Using the mean score of the three co-investigators, there was a small but statistically significant improvement in scores for the final versus the original version (mean difference 1.10 points on the 0–100 points scale; 95% CI: 0.54–1.66). Differences in scores for each co-investigator are presented in Figure 2. Of note, the external rater gave a lower score to the final version of the abstract in 10/72 (14%) evaluated pairs.

None of the independent variables was statistically associated to the improvement in scores (Table 2). However, there was a pattern toward a higher impact of ChatGPT for researchers being less comfortable in English (P = 0.062) and for those who had less experience in abstract submission (P = 0.085).

**Table 2.** Association between independent variables and difference in score (final − original version) as assessed by the participants

Variables	Mean difference	P-value on ANOVA
Type of study		
Quantitative	7	0.282
Qualitative	11	
Other	9	
Presence of table/figure	7	0.712
No. of table/figure	8	
Researchers’ information		
Age group		
18–24 years	12	0.779
25–34 years	8	
35–44 years	7	
45–54 years	7	
55–64 years	10	
Gender		0.360
Female	9	
Male	7	
Number of previous abstracts		0.085
This is the first	20	
1–5	9	
6–15	8	
>15	6	
ChatGPT previous experience		0.856
Never used it	8	
Used it a few times	8	
Used it many times	10	
English literacy		
Moderately comfortable	11	0.062
Quite comfortable	8	
Very comfortable	5	

## DISCUSSION

This experimental prospective study demonstrated that the use of ChatGPT-4 was associated to an improvement in abstract quality, as self-evaluated by 24 international researchers. The mean difference of 8.0/100 points in abstract scores between the final and original versions suggests a substantial enhancement. ChatGPT-4 could contribute positively to the refinement of scientific abstracts by providing suggestions that could be incorporated in the abstract.

To our knowledge, this is the first study evaluating the impact of the use of artificial intelligence to improve abstract writing. As mentioned, Gao et al. demonstrated that human can difficultly discriminate real abstracts from those generated by ChatGPT (24). However, they did not compare the quality of the abstracts. On the other hand, Khlaif et al. conducted an experimental study asking ChatGPT to produce 4 articles and 50 abstracts. They did not provide results related to the quality of the abstracts but concluded that ChatGPT can “improve the quality of high-impact research articles” (27).

The observed improvement in abstract quality seemed consistent across various demographics and experience-related variables. However, while none of these factors reached statistical significance, there was a trend toward a higher impact of ChatGPT for researchers who were less comfortable in English and those with less experience in abstract submission. This results align with Del Giglio et al. who suggested that artificial intelligence could improve scientific writing of non-native English-speaking scientists (28). It may also be helpful for less experienced researchers.

Our primary goal was to evaluate whether ChatGPT could assist researchers in enhancing their medical abstracts. Rather than using a dichotomous question (yes or no) to ask participants if they felt ChatGPT improved the quality of their abstracts, we quantified this by having them rate their own abstracts. While the ChatGPT version of the abstracts tended to have the lowest score, the final abstracts consistently surpassed the originals, suggesting that researchers used some parts of the ChatGPT version to improve their final version. The use of ChatGPT could be seen as an external revision. The researchers’ discomfort with submitting the ChatGPT-generated abstracts for conference consideration is also a notable finding. Among the participants, 75% reported feeling uncomfortable using the ChatGPT version for submission, primarily due to omissions of critical information. This suggests that, despite the initial output, researchers played a crucial role in refining and improving the abstracts, leveraging ChatGPT as a valuable tool in the revision process.

The study must be put in the context of its limitations. Foremost among these limitations is the unblinded nature of the primary outcome measurement. This deliberate choice was motivated by our desire to directly ask researchers whether ChatGPT had a discernible positive impact and to quantify such perceived benefits. The subsequent blinded evaluation conducted by the three co-authors validated the enhancements observed in the final version. Even though 75% of the researchers reported that the use of ChatGPT improved their final abstract, we were not able to measure the influence of external factors to improve the final abstract. For example, it is possible that having another look at the abstract 24 hours after releasing it permitted to improve some aspects of the abstract. We did not measure the acceptance rate of the abstracts. Ideally, we would have submitted both versions to the conference website and compared acceptance rates, but such a comparative analysis was deemed ethically untenable. Our sample size was rather small and limited our power to identify the impact of ChatGPT among different subgroups. Finally, this was a convenience sample, and it is possible that participants may have overestimated the impact of ChatGPT.

## CONCLUSION

In conclusion, our study demonstrates evidence supporting the potential of ChatGPT to help researchers enhance the quality of scientific abstracts. This may be particularly relevant for researchers facing barriers to abstract generation including experience and language. While the tool has shown promise in helping researchers, vigilance is crucial to address factual errors and identify omission. As artificial intelligence continues to evolve, understanding its role in the scientific writing process becomes increasingly important.
